# Multifocal Retinocytoma Associated With Intronic Acceptor Splice Site Variants in the RB1 Gene

**DOI:** 10.7759/cureus.70786

**Published:** 2024-10-03

**Authors:** Jennifer Li-Wang, Patricia Chévez-Barrios, Jessica S Thomas, Amy C Schefler

**Affiliations:** 1 McGovern Medical School, UTHealth Houston, Houston, USA; 2 Pathology and Laboratory Medicine, and Ophthalmology, Weill Medical College of Cornell University, New York City, USA; 3 Ophthalmology, Baylor College of Medicine, Houston, USA; 4 Ophthalmology, Head and Neck, University of Texas MD Anderson Cancer Center, Houston, USA; 5 Pathology and Genomic Medicine, Houston Methodist Hospital, Houston, USA; 6 Pathology and Laboratory Medicine, Weill Medical College of Cornell University, New York City, USA; 7 Pathology and Laboratory Medicine, Houston Methodist Hospital, Houston, USA; 8 Clinical Ophthalmology, Weill Medical College of Cornell University, New York City, USA

**Keywords:** retinoblastoma, retinocytoma, retinoma, subretinal seeds, vitreous seeds

## Abstract

Retinocytomas are benign tumors that arise from mutations in the *RB1* gene. Previous research describes the appearance of retinocytomas as that of treated retinoblastoma (Rb) lesions, with characteristics such as chorioretinal atrophy, calcification, and a lack of necrosis or mitotic activity on histopathology. We present the unusual case of an asymptomatic seven-year-old girl with two independent translucent masses in the peripheral retina of the right eye (OD) and extensive intraretinal tumor and vitreous seeds. Initial fundus examination and B-scan ultrasonography documented the two lesions with extensive placoid intraretinal tumor, uncalcified vitreous seeds, and an area of large subhyaloid seed on the optic nerve (ON) head. Given the clinical appearance and elevated intraocular pressure (IOP), the eye was staged as Group E and enucleated. Histopathology performed on the enucleated specimen revealed pure retinocytoma with two predominant retinal tumors, extensive flat intraretinal tumor, large subhyaloid seeds over the inner limiting membrane of the ON and focally central retina and localized uncalcified vitreous seeds with no malignant Rb component. A next-generation sequencing (NGS) panel detected two intronic acceptor splice site variants in the *RB1* gene. While the previous literature documents cases of retinocytoma with vitreous seeds, this is the first case to our knowledge of a sporadic multifocal retinocytoma with a large ON prelimiting membrane, subhyaloid seeds, and vitreous seeds associated with two intronic acceptor splice site variants in the *RB1* gene and no other detectable mutations.

## Introduction

Retinoblastoma (Rb) is the most common primary intraocular cancer in children, often characterized by the presence of a translucent retinal mass in one or both eyes [1]. The majority of Rb tumors result from mutagenesis in the *RB1* gene, although a small percentage of cases may be related to amplification of the MYCN proto-oncogene [2]. Rb may arise in an autosomal dominant inheritance pattern (30%-40% of cases) or sporadically (60%-70% of cases) [1]. In the United States, the vast majority of Rb cases are diagnosed in children under five years old. The International Intraocular Retinoblastoma Classification (IIRC) system is the current standard for classifying Rb, staging tumors from A to E (with the highest rates of globe salvage for eyes in groups A through C) [3]. Of note is the extent of vitreous and subretinal seeding at presentation, which is an important factor in staging Rb.

Retinocytoma (or retinoma) is considered a benign retinal neoplasia of an *RB1* gene mutation. Initially considered a spontaneously regressed Rb, retinocytoma is now theorized to be a pre-malignant lesion that may later accrue additional genetic mutations and undergo malignant transformation into an Rb [4]. One study reported that the estimated risk of malignant transformation for a retinocytoma was 4%, but this study was limited by sample size and provider bias [5]. Classic clinical findings of retinocytomas include a translucent retinal mass, chorioretinal atrophy, retinal pigment epithelium (RPE) changes, and calcification [5]. Previous literature describes the appearance of retinocytomas as that of “spontaneously regressed” or “treated” retinoblastomas [5], although retinocytomas with atypical features such as vitreous seeds have been reported [6-9]. Malignant transformation of a retinocytoma into an Rb has been documented in rare cases [10], but the complete spontaneous differentiation of Rb into retinocytoma without atrophic or necrotic retinal changes has not been described.

Histopathological features characteristic of retinocytomas include well-differentiated tumor cells with photoreceptor differentiation arranged in fleurettes or with neuronal differentiation. Retinocytoma cells do not demonstrate any evidence of necrosis or mitotic activity [11]. As direct tumor biopsy is generally contraindicated in cases of suspected Rb, histopathology is typically only performed on enucleated eyes, making clinical judgment paramount to the diagnosis.

## Case presentation

A healthy, asymptomatic seven-year-old girl presented with lesions in the peripheral retina of the right eye (OD). The lesions were incidentally discovered by an optometrist, who noted abnormal obscuration of the optic nerve (ON) on a routine exam. The patient was then referred to ocular oncology by a pediatric ophthalmologist.

**Figure 1:**
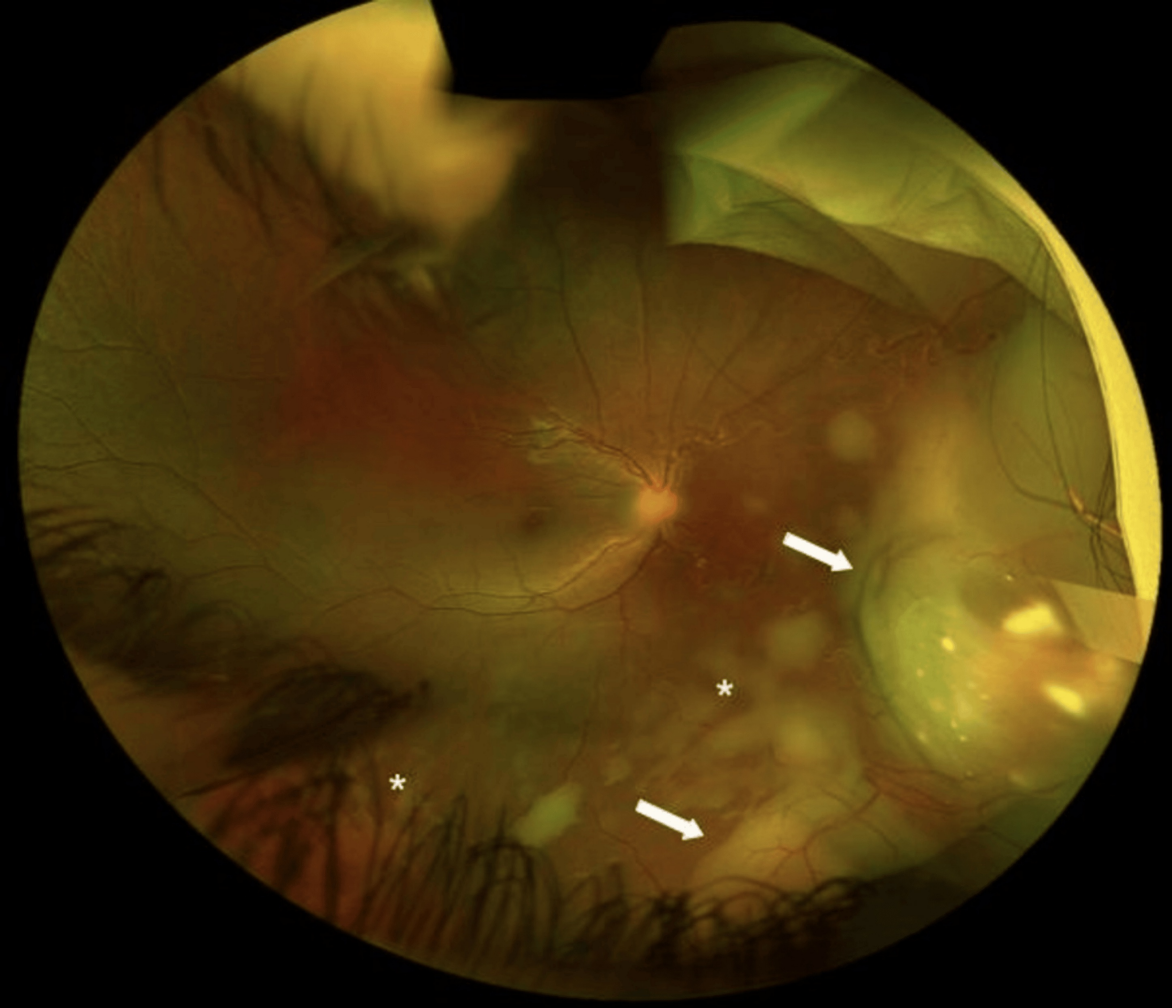
Fundus photograph montage of retinal tumors Fundus photograph of the right eye shows two translucent retinal masses (arrows) with area suspicious of subretinal seeds (*).

The patient denied past ocular, systemic, and pertinent family history. Visual acuity was 20/20 OD and 20/25 OS. Clinical exam and diagnostic imaging showed two tumors OD: a primary partially calcified tumor temporally and a secondary uncalcified tumor inferotemporally (Figure 1). The primary tumor had surrounding RPE changes. There were extensive retinal nodular tumors suspicious for subretinal seeding between the tumors and the ON.

**Figure 2:**
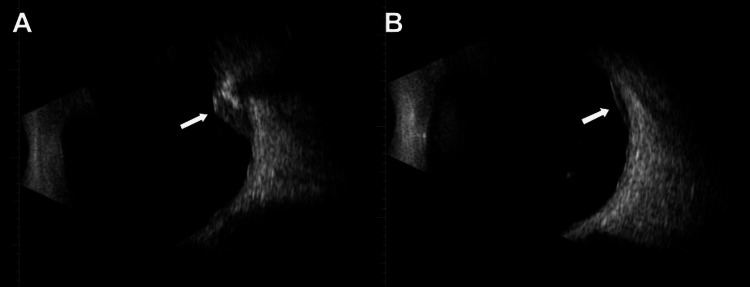
B-scan ultrasound of the right eye showing intraocular lesions (A) Right B-scan ultrasound showing the primary partially calcified lesion (arrow). (B) Right B-scan ultrasound showing the secondary uncalcified lesion (arrow).

B-scan measurements of the primary partially-calcified tumor were 11.0 by 12.5 millimeters (mm) in base and 4.5 mm in height (Figure 2A). The secondary tumor measured 7.0 by 6.5 mm in base and 1.2 mm in height (Figure 2B).

**Figure 3:**
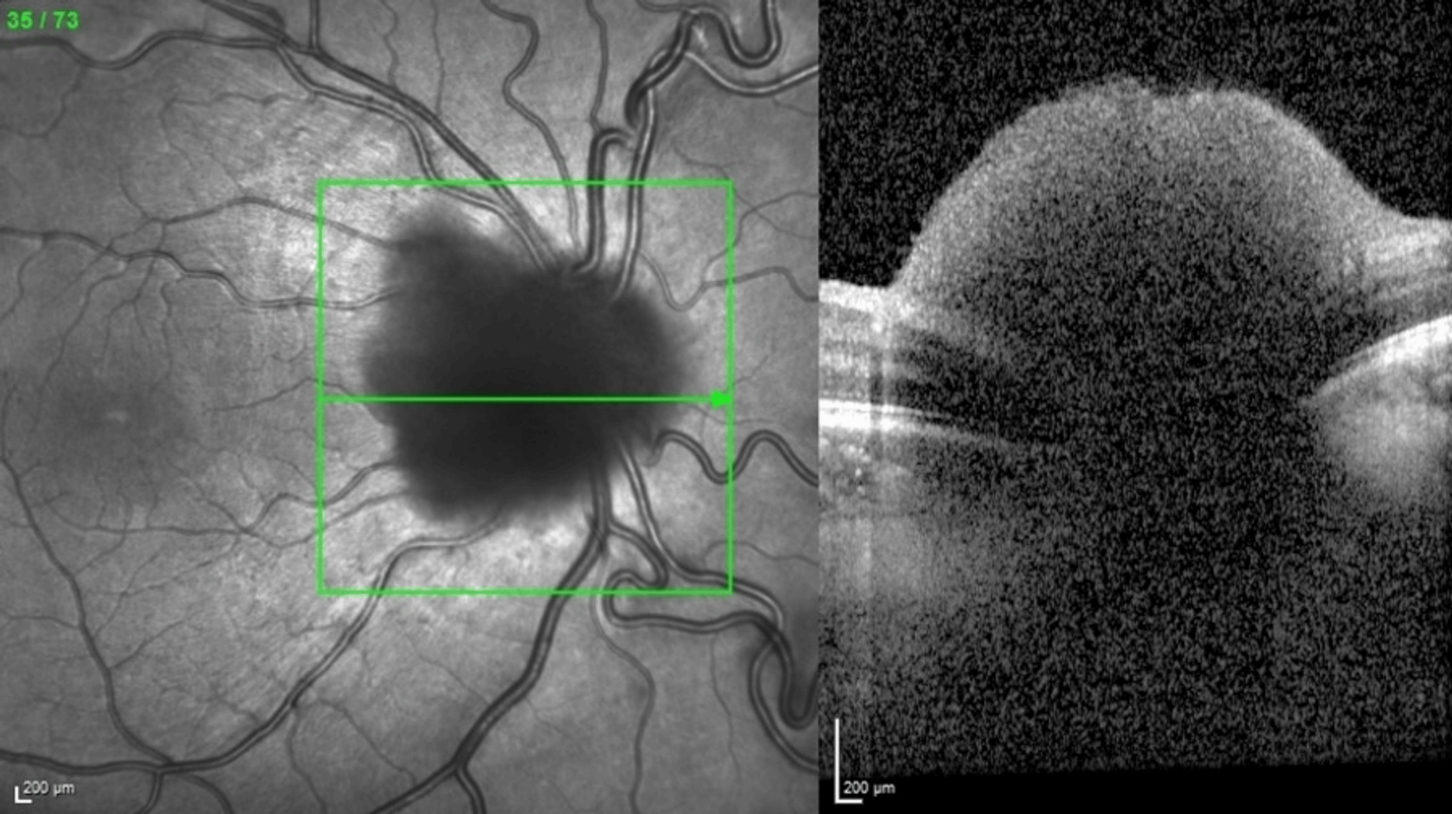
(Left, right) Enhanced depth imaging spectral-domain optical coherence tomography (EDI-OCT) over the right optic nerve showing subhyaloid seeds on top of the optic nerve head.

In addition to the two tumors, enhanced depth imaging spectral-domain optical coherence tomography (EDI-OCT) showed subhyaloid seeds on top of the right ON head (Figure 3). The left eye (OS) was normal.

Exam under anesthesia (EUA) the next day confirmed the presence of the initial lesion, diffuse infiltration presumed to be subretinal seeds posteriorly and temporally, and rare vitreous seeds. Ultrasound biomicroscopy (UBM) demonstrated small seeds touching the anterior lens capsule. The patient was diagnosed with a Group E Rb. Given the elevated intraocular pressure (IOP) (32 by pneumotonometer during EUA), atypical appearance of the lesion, and ON tumor location, the decision was made to perform an enucleation.

Given the late presentation, a full metastatic workup was performed (i.e., bone marrow biopsy, magnetic resonance imaging (MRI) of the brain and orbits, lumbar puncture with cytology). For *RB1* germline testing, sequencing of the RB1 gene was performed on peripheral blood.

**Figure 4:**
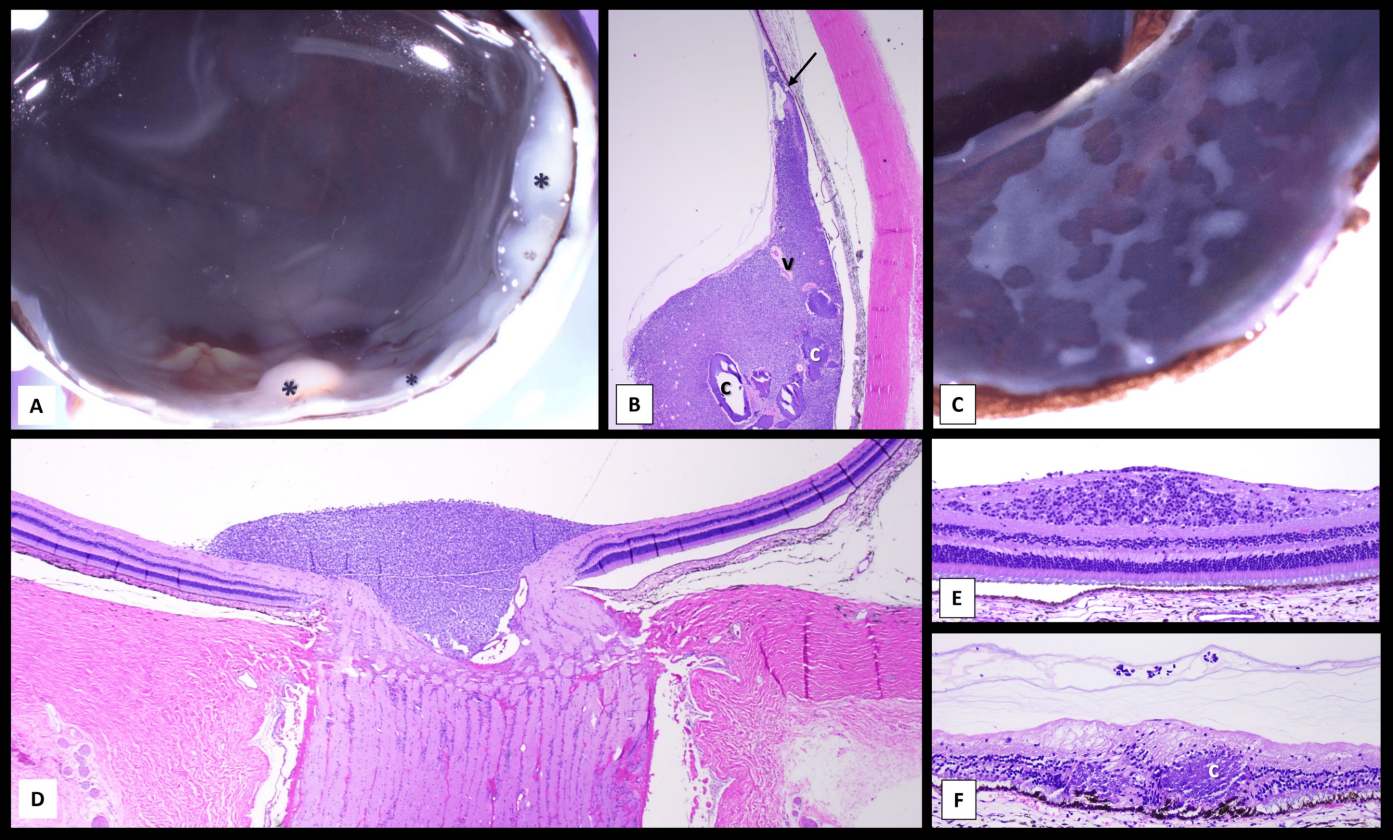
Pathology features of the multifocal tumors (A) Macroscopic view of the posterior and equatorial retina and optic nerve containing multifocal white-gray translucent-appearing tumors in the nasal and superior quadrants and the head of the optic nerve (*). The macula is uninvolved (yellow with artificial puckering in the back). (B) Histologic view of the largest intraretinal tumor located in the equator and extending into the ora serrata (arrow). Notice the large areas of calcification (c) and prominent vessels (v) (hematoxylin and eosin, original magnification 1.25×). (C) Macroscopic view of the superior nasal calotte with a variegated, geographic, white-gray appearance of the intraretinal tumors suggestive of subretinal seeding. Macroscopic and microscopic examination shows no subretinal seeding but instead extensive intraretinal outer and inner tumors. (D) Panoramic view of the optic nerve and peripapillary retina with the tumor on the surface of the optic nerve head without significant invasion into the optic nerve neural tissue at any level. The tumor molds into the optic nerve cup (hematoxylin and eosin, original magnification 2×). (E) Retina with expansion to the inner nuclear layers by the retinocytoma with associated epiretinal tumor seeding (hematoxylin and eosin, original magnification 4×). (F) A separate peripheral focus of intraretinal tumor mostly in outer layers with calcification and RPE proliferation into the tumor (c). Notice the condensed vitreous bands containing few tumor seeds (hematoxylin and eosin, original magnification 4×).

**Figure 5:**
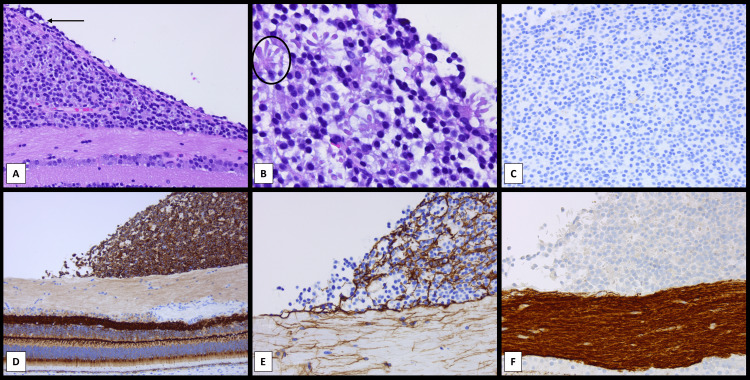
Histopathologic characterization of the retinocytomas (A) The tumor is composed of very well-differentiated tumor forming either fleurettes or areas of neuronal differentiation without necrosis or mitosis. Most of the tumor is intraretinal with few areas of tumor cells extending into the vitreous and over the inner limiting membrane (arrow) (hematoxylin and eosin, original magnification 10×). (B) The tumor shows many areas of photoreceptor differentiation with fleurettes (circle) (hematoxylin and eosin, original magnification 40×). (C) The tumor shows no proliferation demonstrated here by the absence of staining with Ki67 (Immunohistochemistry using Ki67 (Mib1) antibody, DAB chromogen in brown and hematoxylin nuclear counterstain, original magnification 20×). (D) The tumor cells stain uniformly with synaptophysin on the surface of the peripapillary retina. Synaptophysin highlights the synaptic areas of the retina and the outer segments of the cones (Immunohistochemistry using synaptophysin antibody, DAB chromogen in brown and hematoxylin nuclear counterstain, original magnification 10×). (E) A meshwork of glial cells is highlighted by the glial fibrillary acidic protein in the inner retinal layers extending to the tumor. Those cells in the vitreous lack the glial meshwork. The nerve fiber layer of the retina shows an increased number of reactive astrocytes (Immunohistochemistry using glial fibrillary acidic protein (GFAP) antibody, DAB chromogen in brown and hematoxylin nuclear counterstain, original magnification 20×). (F) The nerve fiber layer of the retina is highlighted by the neurofilament stain and shows the tumor cells over the inner limiting membrane in this peripapillary retina (Immunohistochemistry using neurofilament antibody, DAB chromogen in brown and hematoxylin nuclear counterstain, original magnification 20×).

Histopathology performed on the enucleated tissue revealed the two large lesions, extensive multifocal tumors, and vitreous seeds, but no true subretinal seeds or malignant Rb component (Figures 4A-4F). The tumor composition consisted mostly of photoreceptor differentiation (fleurettes) and areas of single and groups of cells with neuronal differentiation (Figures 5A-5F). No apoptosis, mitosis, or necrosis was noted, confirmed by the absence of staining with the Ki67 (Mib1) proliferation marker via immunohistochemistry (IHC). Vitreous seeds were composed of detached fleurettes or small round cells with neuronal differentiation. On the ON, tumor cells filled the nerve cup over the inner limiting membrane (ILM) without invasion into the axonal nerve.

Interestingly, the tumor over the ILM in the peripapillary retina and the ON showed a delicate stroma-like meshwork of glial cells migrating from the inner layers of the retina. This suggests the tumor has been established in these areas for a prolonged period of time (Figures 5A-5F). The apparent subretinal seeds were in fact multifocal intraretinal tumors that occupied most of the retina including expansion of the photoreceptor layer, which gave the clinical impression of subretinal seeding (Figures 4, 5). No choroidal or retrolaminar invasion was noted. The vitreous posterior to the lens and the trabecular meshwork contained rare macrophages.

Next-generation sequencing (NGS) performed on the formalin-fixed paraffin embedded tissue that included the *RB1* gene detected two mutations: RB1:ENST00000267163.4:c.607+1del and RB1:ENST00000267163.4:c.940-2A>G. Both of these are intronic acceptor splice site variants in the *RB1* gene and have been previously reported as pathogenic for Rb. The metastatic workup returned within normal limits. Germline genetic testing returned negative for an *RB1* mutation.

## Discussion

This is the first case to our knowledge to describe a retinocytoma with extensive subhyaloid and vitreous seeding associated with intronic *RB1* variants. The subhyaloid seeds on the ON head were concerning for potential invasive postlaminar ON extension and were previously undocumented findings in an eye with retinocytoma.

Four studies described six patients with vitreous seeding associated with retinocytomas. Of these six patients, two had localized calcified vitreous seeds [6,7], one had diffused calcified vitreous seeds [9], and two had localized vitreous seeds with no comment on their calcification status [8]. In our case, localized uncalcified vitreous seeds were noted on exam and histopathology. The presence of diffuse subretinal or vitreous seeding extending greater than 3 mm from the tumor is a clinical characteristic of advanced Rb according to the IIRC. In our patient, the uncalcified vitreous seeds and subhyaloid seeds presented an aggressive clinical picture, and subsequent histopathology showed these to be composed of retinocytoma, which is a previously undocumented finding.

The clinical and histopathological findings suggest two possibilities for tumor development. The lesions were likely either (a) multifocal de novo retinocytomas driven by the chromosomic variants that act on the mRNA splicing mechanism promoting an aberrant protein formation or (b) Rb lesions that arrested during a period of activity and redifferentiated into retinocytomas. The latter theory is less likely, as there are no residual areas of necrosis or atrophy. Taking into consideration that the tumor is multifocal in an older patient, there is a strong possibility that this patient has somatic low-level mosaicism. Sporadic, de novo low-level mosaicism involving the *RB1* gene may not be detected in the blood with most sequencing techniques. In some cases of low-level mosaicism (~19%), the mutation is still not detected in blood even after ultra-deep NGS [12].

Pre-messenger RNA (pre-mRNA) is modified via splicing to produce mature mRNA. Splicing removes introns and connects encoding exons to produce specific proteins. This process occurs during or shortly after transcription. Genes with splice site variants may affect the production of mRNA and the final protein in different ways, depending on the location and nature of the specific alteration. Splice site mutations may result in a dysfunctional protein. Recently in breast cancer, some naturally occurring alternative transcripts of *BRCA1* and *BRCA2*, through mutational variants in the splicing sites, have been found to encode protein isoforms with residual tumor suppressive activity [13]. Combined genetic and splicing analysis of *BRCA1* c.[594-2A>C; 641A>G] highlights the relevance of naturally occurring in-frame transcripts for developing disease gene variant classification algorithms [14]. Therefore, for genetic counseling and understanding of *BRCA* gene mutations, the pathogenic potential may be significantly less than assumed for the predicted loss-of-function variants located in an exon that is not present in these alternative transcripts.

In the present case, these two intronic *RB1* variants may result in the production of a dysfunctional Rb protein that allows for arrested differentiation of the tumor. These exact mutations identified in this patient have not been associated with retinocytoma before but may explain the unique phenotype of the tumors.

## Conclusions

This case is the first to our knowledge of a unilateral, multifocal retinocytoma with vitreous seeds, subhyaloid seeds over the ON, and unique intronic acceptor splice site variants in the *RB1* gene. Clinicians should be aware that retinocytomas with no active apoptosis or necrosis on pathology can appear uncalcified and can demonstrate clinical features that are typically associated with more advanced retinoblastoma, such as vitreous seeds, subhyaloid seeds, and subretinal seeds. In this case, it was impossible to determine clinically that these cells were not actively dividing and thus enucleation was necessary. Furthermore, attempts at globe salvage such as intra-arterial chemotherapy would likely not change the fundus appearance of this patient, making both diagnosis and treatment challenging.
